# Placing Health Warnings on E-Cigarettes: A Standardized Protocol

**DOI:** 10.3390/ijerph15081578

**Published:** 2018-07-25

**Authors:** Jennifer R. Mendel, Marissa G. Hall, Sabeeh A. Baig, Michelle Jeong, Noel T. Brewer

**Affiliations:** 1 Lineberger Comprehensive Cancer Center, University of North Carolina, Chapel Hill, NC 27599, USA; jrmendel@unc.edu (J.R.M.); mghall@unc.edu (M.G.H.); mjeong@email.unc.edu (M.J.); 2 Department of Health Behavior, Gillings School of Global Public Health, University of North Carolina, Chapel Hill, NC 27599, USA; sbaig1@live.unc.edu

**Keywords:** e-cigarette, warning, constituent, chemical, messaging, addiction

## Abstract

Health warnings for e-cigarettes are a promising and novel tobacco control intervention for reducing e-cigarette use. We developed a new protocol for evaluating e-cigarette warnings by placing them on users’ own devices to reflect real-world exposure. Study 1 participants were a national convenience sample of 606 U.S. adult e-cigarette users surveyed online in March 2017. Most Study 1 participants were willing to have their e-cigarette devices (87%) and refills (83%) labeled. Study 2 participants were a convenience sample of 22 adult e-cigarette users recruited in California, United States in April 2017. We applied the U.S. Food and Drug Administration’s proposed e-cigarette warning to users’ own devices and refills. Most Study 2 participants (81%) reported using e-cigarette devices with our warning labels at least 90% of the time during the study. Nearly all (95%) said they would participate in the study again, and 100% would recommend the study to a friend. Conversations about e-cigarette harms, conversations about quitting e-cigarettes, and intentions to quit using e-cigarettes increased during the study (all *p* < 0.05). These studies show that our naturalistic labeling protocol was feasible, acceptable to participants, and had high retention over three weeks. Using the protocol can yield important evidence on the impact of e-cigarette warnings to inform tobacco warning policies.

## 1. Introduction

Increasing numbers of U.S. youth and adults have tried electronic nicotine delivery systems (or e-cigarettes) [1,2,3]. Many current cigarette smokers regularly use e-cigarettes [4,5] and often believe e-cigarettes can help them quit smoking [2,6]. E-cigarettes may help smokers quit smoking when used with clinical supervision [7,8], but they may hinder cessation or even encourage initiation when used naturalistically [9,10,11]. As a result, sustained dual use of both electronic and combustible cigarettes has increased [12,13]. While the general consensus is that e-cigarettes cause less harm overall than cigarettes [14,15], a growing body of research indicates evidence of health harms from e-cigarette use [16]. For instance, exposure to toxic chemicals via inhalation of e-cigarette aerosol may lead to respiratory impairment [9,17], among other health risks [18,19].

Health warnings for e-cigarettes are a novel and timely tobacco control intervention that hold promise for discouraging inappropriate e-cigarette use. The U.S. Food and Drug Administration (FDA) recently extended its authority to include e-cigarettes and to require a single text-only warning about addiction on packaging and advertisements for e-cigarettes that contain nicotine [20]. A large and growing body of research indicates that cigarette pack warnings help smokers quit [21,22]. However, research is needed to understand the effects of e-cigarette warnings on intended (e.g., discouraging e-cigarette use) and unintended consequences (e.g., increasing cigarette use) [23]. Online experiments have found that brief one-time exposures to warnings about e-cigarette harms increase thoughts about not using e-cigarettes, while reducing positive evaluations of and intentions to purchase e-cigarettes [24,25,26]. Two qualitative studies have highlighted the complexity of developing warnings for e-cigarettes, further pointing toward a need for additional studies with diverse populations [27,28]. However, no studies have assessed the impact of actually placing warnings on users’ e-cigarettes for several weeks, an intervention with the potential for high exposure and reach that pairs the warning with behavior. Tobacco control researchers would benefit from a naturalistic method for evaluating the FDA’s proposed warnings and other e-cigarette warnings that replicate real-world exposure to the warnings [29].

Two large randomized controlled trials have successfully used the University of North Carolina (UNC) tobacco product warning protocol [29] to evaluate the impact of placing new messages on smokers’ own cigarette packs for several weeks (total *n* = 2868) [21,30]. In the protocol, smokers bring a supply of cigarette packs to weekly appointments; then, study staff label the cigarette packs while smokers take a survey [29]. In the current proof of principle study, we aimed to adapt the UNC protocol for evaluating warnings for e-cigarette devices and refills. In two pilot studies, we separately examined e-cigarette users’ initial receptivity to the proposed protocol and their satisfaction with the actual protocol. To further validate the use of warnings as a viable tobacco control intervention for reducing e-cigarette use, we also aimed to assess short-term changes in both intended and unintended outcomes.

## 2. Study 1: Acceptability

The aim of Study 1 was to gauge initial reactions from e-cigarette users about the acceptability of participating in an e-cigarette labeling study and to determine how to modify the UNC protocol [29] for e-cigarettes.

### 2.1. Materials and Methods

#### 2.1.1. Participants and Procedures

In March 2017, we recruited a convenience sample of 1350 U.S. adults (18+) through Amazon Mechanical Turk (MTurk), an online platform commonly used for behavioral and social science research [31,32,33,34,35]. The current study was part of a larger parent study that set a sampling quota for 900 cigarette smokers and 450 non-smokers. For the current study, we report data for 606 participants who were current e-cigarette users (i.e., used e-cigarettes every day or some days). After providing informed consent, participants completed an online questionnaire. Participants received US $1.25 after completing the brief survey. The UNC institutional review board approved this study (study number 17-0133).

#### 2.1.2. Measures and Analysis

The survey assessed standard demographics and combustible cigarette use. We defined being a current smoker as having smoked at least 100 cigarettes in one’s lifetime and now smoking every day or some days, and defined being a former smoker as having smoked at least 100 cigarettes in one’s lifetime but not smoking currently. The survey also measured whether participants attempted to quit using e-cigarettes (defined as having stopped using for one day or longer in the past week because they were trying to quit using e-cigarettes), adapting the standard quit attempt item for combustible cigarettes [36]. The survey then briefly described an e-cigarette warning protocol and study: “Imagine you have been recruited to participate in a new research study. In this study, you would come to the research office every week for four weeks, and a sticker with a health warning would be put on your e-cigarette. During the study, you’d use the labeled e-cigarette as you normally would. You would get up to $300 for being in the study.” Finally, the survey assessed whether participants would be able to bring in an eight-day supply of refills and willing to have their e-cigarette device, package, refills, and refill package labeled. Response options were “yes”, “no”, and “not sure”. Survey measures appear in Supplementary Materials (Tables S1 and S2). We calculated descriptive statistics to summarize demographic information and process measures and used logistic regression to examine demographic predictors of process outcomes, using Stata/SE version 14.1 [37].

### 2.2. Results

About half of Study 1 participants (45%) were aged 29 or younger (Table 1). Almost all participants (92%) were current smokers. Average e-cigarette use frequency was three days in the past week and average intensity was 41 puffs on the days they used e-cigarettes. Fourteen percent had made an e-cigarette quit attempt of at least 24 h in the past week.

**Figure 1:**
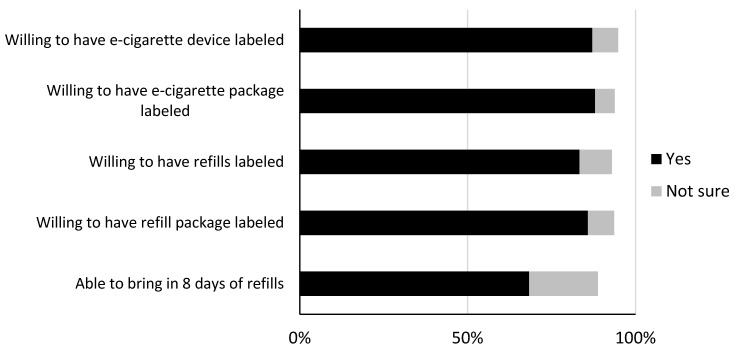
Acceptability of e-cigarette warning protocol, Study 1 (*n* = 606). Missing data ranged from 0% to 1.0%.

Most participants (85%) owned and regularly used only one or two e-cigarette devices. The most common types of refills used were e-liquid poured in a tank (45%) and pre-filled cartridges (32%); 8% used disposable e-cigarettes that do not have refills. Most (68%) said they would be able to bring in an eight-day supply of refills, and 21% said they were not sure. Most said they would be willing to have their e-cigarette device (87%), device package (88%), refills (83%), and refill package (86%) labeled (Figure 1). E-cigarette users who vaped more frequently had higher odds of being willing to have their e-cigarette package (OR = 1.21), refills (OR = 1.17), and refill package (OR = 1.16) labeled, and higher odds of being able to bring in eight days’ worth of refills (OR = 1.26, all *p* < 0.05). E-cigarette users who were younger (OR = 0.60) and had tried to quit e-cigarettes in the past week (OR = 0.47) were less likely to report being able to bring in eight days’ worth of refills (both *p* < 0.05). Otherwise, the protocol was equally acceptable across populations.

### 2.3. Discussion

Most e-cigarette users in Study 1 were willing to have their e-cigarette devices and refills labeled with a health warning. Most also reported being able to bring in enough refills to participate in a four-week labeling study. E-cigarette users were receptive to the warning protocol as we described it and would be likely to enroll in an e-cigarette labeling study. Importantly, the warning protocol was acceptable across diverse groups, and e-cigarette users who vape more frequently were particularly receptive to the protocol. The results of Study 1 informed two decisions regarding the protocol for Study 2. First, we decided to instruct participants to bring in one or two e-cigarette devices they own and use most frequently for labeling based on the finding that few e-cigarette users used more than two devices. Second, we decided to allow e-cigarette users to bring in disposable e-cigarettes for labeling in Study 2 since a small but meaningful proportion of e-cigarette users in Study 1 reported using disposable e-cigarettes.

## 3. Study 2: Feasibility

The goal of Study 2 was to test our UNC tobacco product warning protocol [29] modified for e-cigarettes. Specifically, we examined the feasibility of recruiting and retaining e-cigarette users, as well as the feasibility of placing warnings on their devices and refill materials. We also explored whether e-cigarette-related psychosocial outcomes changed during the study. Finally, we aimed to develop best practices for putting warnings on e-cigarette devices and refill materials.

### 3.1. Materials and Methods

#### 3.1.1. Participants

In April 2017, we recruited 22 adult e-cigarette users in the Bay Area in California, US. Eligibility criteria were being 21 years or older, being a current e-cigarette user (defined as now using every day or some days), using an e-cigarette that contains nicotine, being able to attend three weekly appointments, being able to bring in e-cigarette devices and eight days’ worth of refills to each visit for labeling with warnings, being able to complete a paper and pencil survey without help, and being able to speak English. We excluded pregnant women and participants whose e-cigarette devices could not be labeled (e.g., if the label did not stick on device material). We recruited participants through Craigslist and screened potential participants for eligibility online and by phone.

#### 3.1.2. Procedures

We adapted the UNC protocol [29] for placing warnings on e-cigarettes and refills, as described below. We invited participants to attend three visits, each spaced one week apart, at the study office in San Francisco, California, US. At Visit 1, we confirmed eligibility, obtained written informed consent, and enrolled e-cigarette users. Participants completed a paper survey at all three visits.

**Figure 2:**
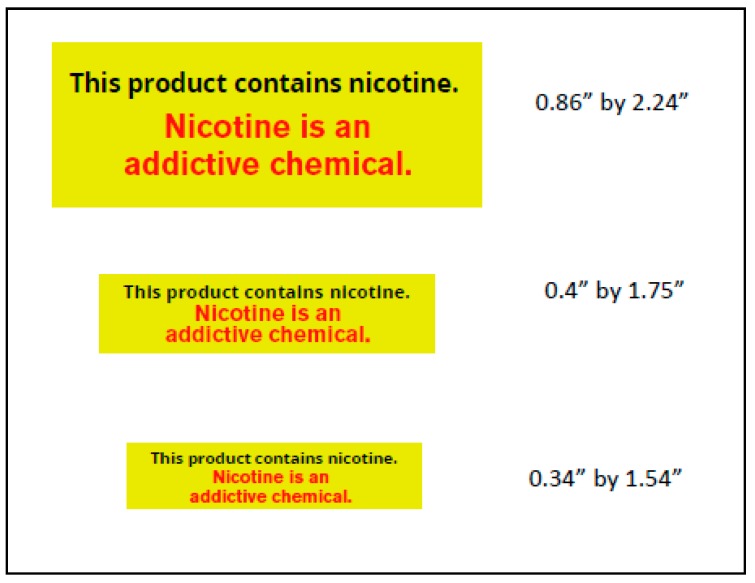
Warning labels for e-cigarette devices and refills, Study 2.

We asked participants to bring in up to two e-cigarette devices they own and regularly use to Visits 1–3. We also asked participants to bring in eight days’ worth of e-cigarette refills to those visits; users of disposable e-cigarettes were instructed to bring eight days’ worth of disposable e-cigarettes. In front of the participant, research staff applied a self-adhesive label with the warning message to the participant’s e-cigarette devices and refills at Visit 1. The message was adapted from the FDA warning that will be required on packaging and advertisements of e-cigarettes that contain nicotine: “WARNING: This product contains nicotine. Nicotine is an addictive chemical” [20]. We used the message verbatim except we removed the marker “warning” so the text could be larger and thus more easily read (Figure 2). Warning labels came in three sizes and study staff used the largest label that fit on the device, applying labels to the e-cigarette on the area farthest from the mouth piece. Study staff also labeled e-cigarette refills when possible, but some of the refills were too small to label. Study staff made notes about the labeling process to help inform best practices.

At Visit 2, study staff applied warning labels to new devices and refills as needed. At the end of each visit, on completion of the survey, participants received a cash incentive that totaled up to $200 across the study. At the end of the study, we offered participants information and resources about tobacco cessation. None of the participants withdrew from the study. The UNC institutional review board approved this study (study number 13-2430).

#### 3.1.3. Measures and Analysis

Surveys used validated measures adapted from prior studies to assess constructs from the UNC Tobacco Warning Model [38], including noticing the warning [39], social interactions about the warning [40,41], thinking about the information in the warning [42,43], intention to quit using e-cigarettes (three items) [44], number of times forgoing using an e-cigarette in the past week [45,46], attempting to quit using e-cigarettes for one day or longer in the past three weeks [36], and quitting e-cigarettes (i.e., did not use an e-cigarette in the past seven days as of the end of the study) [47]. Additionally, the surveys measured worry about the harms of using e-cigarettes (four items) [48,49,50,51], positive e-cigarette user prototypes (four items) [52,53,54], negative e-cigarette user prototypes (four items) [52,53,54], the percentage of the time participants used labeled e-cigarette devices, participant satisfaction with study procedures, and participant demographics. The surveys also assessed combustible cigarette use and the number of days in the past week that participants smoked cigarettes to look at potential unintended consequences of the warning (i.e., increased cigarette consumption). We used the same definitions as Study 1 for smoking status. Survey measures appear in Supplementary Materials. 

Analyses used Stata/SE version 14.1, R (v. 3.3.2) [37] and BayesFactor (v. 0.19.12-2) [55]. We calculated descriptive statistics to summarize participant demographics, process measures, and social interactions about the warning. To assess changes over time, we present means for baseline and the final follow-up visit and difference scores along with 95% confidence intervals from paired samples *t*-tests. We confirmed these results using Bayesian parameter estimation [56,57], an approach that is well-suited to small samples [58]. Bayesian analyses used an uninformative prior (concentration = 1) [59] for e-cigarette quit attempts and an informative Cauchy prior (*r* = $\sqrt{2}/2$) [60] for all other outcomes, reflecting our previous cigarette pack labeling trials that produced small and occasionally medium or larger effects [21,30].

### 3.2. Results and Discussion

About a third of participants (36%) were aged 29 or younger (Table 2). Half of e-cigarette users were white, and 14% were African American. Most (68%) were male and 27% were gay, lesbian, or bisexual. Half of participants were current smokers, and 45% were former smokers. Participants reported using e-cigarettes an average of six days in the past week and taking an average of 38 puffs on the days they used e-cigarettes. Five percent had made an e-cigarette quit attempt of at least 24 h in the past week. Most (90%) owned and regularly used one or two e-cigarette devices.

#### 3.2.1. Process Measures

**Figure 3:**
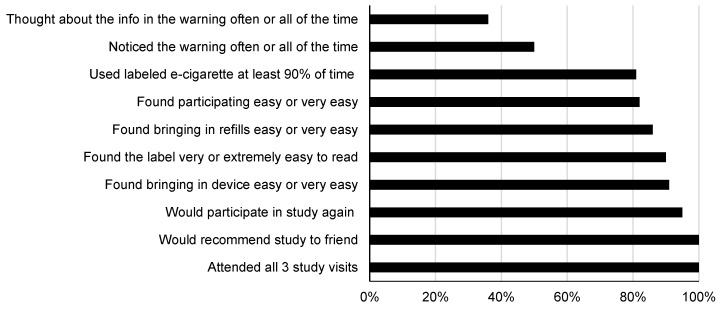
Process evaluation measures for e-cigarette labeling protocol, Study 2 (*n* = 22). *Note.* One participant had missing data for noticing the warning and one had missing data for percentage of the time using a labeled e-cigarette; no other missing process data.

About one-third (36%) of participants said they thought about the information in the warning often or all of the time; and 90% found the label very or extremely easy to read (Figure 3). About half said they noticed the warning often or all of the time. Study participants reported high rates of adherence to using labeled e-cigarettes; 81% used labeled e-cigarettes at least 90% of the time. Many participants (82%) found participating in the study to be easy or very easy, 86% found bringing in refills easy or very easy, and 91% found bringing in e-cigarette devices to be easy or very easy. Nearly all participants (95%) would participate in the study again and 100% would recommend the study to a friend. Retention was 100% for all three visits.

#### 3.2.2. Social Interactions

Most e-cigarette users (82%) talked about the warning with a variety of people during the study. Among the 18 participants who talked about the warning with others, the most common conversation partners were friends (78%), followed by spouses or significant others (50%), co-workers (44%), other family members (33%), people they did not previously know (22%), and health care providers (6%). At the final visit, 13% said their conversations about the warning were mostly negative, 6% were mostly positive, and 81% were somewhere in between. When asked about the content of conversations, most participants reported talking about negative affect elicited by the warning, chemicals in e-cigarette vapor, the health problems caused by using e-cigarettes, and whether the warning would make other e-cigarette users want to quit using e-cigarettes (Table 3).

#### 3.2.3. Changes over Time

Analyses suggested some changes over the course of the study. Between baseline and follow-up, people appeared to have more conversations about the addictiveness of e-cigarettes (mean difference = 0.82, 95% confidence interval (CI) = 0.06 to 1.58) and the health problems caused by using e-cigarettes (mean difference = 0.77, 95% CI = 0.23 to 1.32) (Table 4). Similarly, e-cigarette quit intentions (mean difference = 0.35, 95% CI = 0.06 to 0.64) also appeared to be higher at the end of the study. While initial analyses suggested that the percentage making an e-cigarette quit attempt increased (absolute difference = 24%, 95% CI = 1% to 47%), Bayesian analyses did not confirm this finding. The number of days when participants smoked combustible cigarettes did not appear to increase (mean difference = −0.14, 95% CI = −0.69 to 0.40). At baseline, people smoked combustible cigarettes an average of 2.10 days in the past week, and at follow-up, they smoked an average of 1.95 days in the past week.

## 4. General Discussion

Our proof of principle study of placing warnings on actual users’ e-cigarette devices and refills for an extended period of time suggests that our naturalistic labeling protocol is acceptable to e-cigarette users and feasible to implement. The protocol had high retention and adherence, and participants reported high levels of satisfaction with the procedures. Furthermore, social interactions and e-cigarette quit intentions appeared to have increased over the two-week period of participants using labeled e-cigarettes. Thus, our new e-cigarette labeling protocol offers the potential for studying the impact of e-cigarette warnings with more realistic exposure to warnings compared with lab studies, filling an important gap in the tobacco control literature. Randomized controlled trials could feasibly use the protocol to evaluate the impact of e-cigarette warnings on behavioral outcomes such as e-cigarette quit attempts.

**Figure 4:**
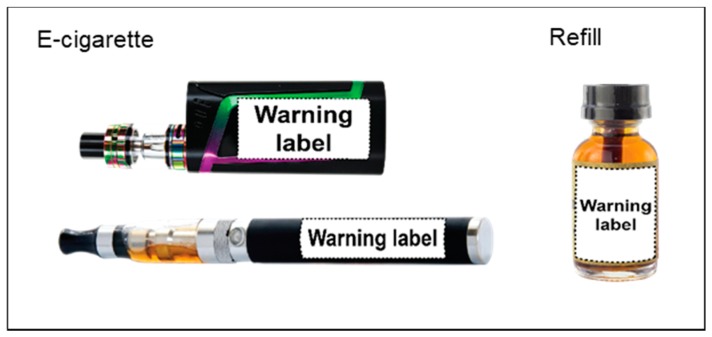
Examples of recommended e-cigarette warning placement.

Adapting the UNC warning protocol [29], we implemented our new e-cigarette warning protocol successfully, and our findings point to six key protocol elements. We recommend an e-cigarette warning protocol that has the steps shown in Table 5: (1) schedule multiple study appointments, spaced one week apart; (2) determine typical e-cigarette consumption through self-report; (3) ask users to bring in their own e-cigarette devices and refills; (4) during study appointments, apply self-adhesive warning labels to users’ devices and refills; (5) provide participation incentives at the end of the appointments; and (6) give participants information about tobacco product cessation services. Based on study staff notes about the labeling process, we offer additional recommendations for warning label design and placement suitable for e-cigarette and refills, as shown in Figure 4. E-cigarette and refill warning labels should be available in several sizes to accommodate variation in product size (e.g., mods vs. vape pens) and study staff should consider selecting the largest size that fits the devices and refills. To maximize readability, study staff should apply the warning labels to participants’ e-cigarette devices lengthwise on the area farthest from mouth piece; refill liquid bottles should be labeled with the message text direction matching that of the bottle. In Study 2, we decided to label the e-cigarette device and refills, rather than the packaging, in order to maximize exposure to the warning. Researchers could adapt the protocol to also place warnings on packaging (e.g., cartridge package or device package) or provide the warning in other ways to enhance exposure (e.g., on a point-of-sale sign).

To evaluate the use of warnings as a viable tobacco control intervention for reducing e-cigarette use, we examined changes in psychosocial and behavioral outcomes. We observed increases in social interactions and e-cigarette quit intentions from baseline to follow-up in Study 2. According to the Tobacco Warning Model [38], social interactions and quit intentions are two key mechanisms by which warnings change behavior. In addition to examining the intended effects of e-cigarette warnings, we examined one potential unintended consequence, finding that the frequency of combustible cigarette use did not appear to increase from baseline to follow-up. Taken together, these findings suggest that e-cigarette warnings hold promise as a tobacco control policy strategy, although we cannot attribute the observed changes solely to the e-cigarette warning because the study did not have a control group. Future studies should examine these outcomes and other possible mediators (e.g., attention, fear, and other negative affect elicited by the warnings [38]) in larger controlled studies. It is possible that the e-cigarette warning we tested and other future e-cigarette warnings could have unintended consequences [61], so future studies should continue to examine a wide range of potential unintended consequences of e-cigarette warnings, such as inaccurate risk perceptions (e.g., the incorrect belief that e-cigarettes are as harmful as or more harmful than cigarettes), increased intentions to switch from e-cigarettes to combustible tobacco products (e.g., cigarettes, hookah), and compensatory increases in combustible tobacco product use [61,62]. Research could inform the possibility of FDA extending the planned single e-cigarette warning on packaging and advertisements to include multiple new warnings and warnings on actual e-cigarette devices and refills [20]. Additional research on e-cigarette warnings could also inform other countries considering implementing this novel tobacco control policy.

The Study 2 sample was small, and thus the study was underpowered to detect changes from baseline to follow-up. We verified results from traditional statistical tests using methods suited to small samples. As stated previously, the absence of a control group means we cannot attribute observed changes solely to the e-cigarette warning. Finally, a limitation of both studies is the use of convenience samples, so the generalizability of findings remains to be established. 

## 5. Conclusions

Better examining the impact of e-cigarette warnings in order to inform tobacco control strategies is a compelling need for public health. Quitting e-cigarettes can reduce exposure to harmful substances, eliminate the maintenance of addiction afforded by dual use, and prevent renormalization of tobacco product use [9]. Our recommended e-cigarette warning protocol developed through this proof of principle study is acceptable and feasible and holds promise as a rigorous strategy for testing new warnings. This protocol maximizes real-world repeated exposures by pairing the warning message with e-cigarette use for greater impact on behavior, while allowing for experimental control necessary to generate rigorous evidence needed to inform U.S. tobacco control policy.

## Figures and Tables

**Table 1:** Participant characteristics, Study 1 (*n *= 606).

Demographics	%
Age (years)	
18–24	16
25–29	29
30–44	42
45–59	11
60+	2
Male	63
Gay, lesbian, or bisexual	10
Race	
Asian	7
Black or African American	7
Other/Multiracial	3
White	83
Hispanic ethnicity	12
Education	
High school diploma or less	14
Some college	31
College degree	49
Graduate degree	7
**Tobacco Product Use**	
Smoking status	
Current smoker	92
Former smoker	5
Never smoker	2
E-cigarette use frequency (days used in past week), mean (SD)	3 (2)
E-cigarette use intensity (puffs on days when used e-cigarette), mean (SD)	41 (93)
Made e-cigarette quit attempt (for 24 h) in past week	14
E-cigarette devices owned and used regularly	
None	5
1 device	61
2 devices	24
3 devices	7
4 or more devices	3
Type of e-cigarette refills used in past 30 days	
E-liquid poured in tank	45
Pre-filled cartridges	32
Disposable e-cigarettes	8
Combination	12
Other	3

SD = standard deviation. Missing data for demographics ranged from 0% to 2.6%.

**Table 2:** Participant characteristics, Study 2 (*n *= 22).

Demographics	%
Age (years)	
21–29	36
30–39	23
40–49	27
50+	14
Mean (SD)	38 (12)
Male	68
Gay, lesbian, or bisexual	27
Race	
Asian	9
Black or African American	14
Other/Multiracial	27
White	50
Hispanic ethnicity	18
Education	
Some college	23
College degree	64
Graduate degree	14
Household income, annual US$	
0–24,999	14
25,000–49,999	45
50,000–74,999	18
≥75,000	23
**Tobacco Product Use at Baseline**	
Smoking status	
Current smoker	50
Former smoker	45
Never smoker	5
E-cigarette use frequency (days used in past week), mean (SD)	6 (1)
E-cigarette use intensity (puffs on days when used e-cigarette), mean (SD)	38 (46)
Made e-cigarette quit attempt (for 24 h) in past week	5
E-cigarette devices owned and used regularly	
1 device	45
2 devices	45
3 devices	9

SD = standard deviation. No missing data for demographic characteristics.

**Table 3:** Content of conversations during the study, Study 2 (*n* = 18).

Content of Conversations	%
Negative affect toward the warning	61
Chemicals in e-cigarette vapor	56
Health problems caused by using e-cigarettes	50
Warning would make other e-cigarette users want to quit using e-cigarettes	50
Warning would stop people from starting to use e-cigarettes	44
This research study	33
Made fun of the warning	28
Warning should be on e-cigarettes	22
Warning makes me want to quit using e-cigarettes	22
Information in this warning is new to me	17

Data from participants who had at least one conversation about the warning; no missing data.

**Table 4:** Changes between baseline and two-week follow-up, Study 2.

	*n*	Baseline Mean (SD)	2-week Follow-Up Mean (SD)	*t*	Difference (95% CI)
Worry	22	2.15 (0.90)	2.35 (0.85)	1.30	0.20 (−0.12 to 0.53)
E-cigarette user prototypes—positive	21	2.06 (0.85)	1.74 (0.70)	−2.01	−0.32 (−0.66 to 0.01)
E-cigarette user prototypes—negative	20	1.63 (0.65)	1.73 (0.71)	0.64	0.10 (−0.23 to 0.43)
No. conversations about the addictiveness of e-cigarettes in past week	22	0.82 (1.79)	1.63 (2.22)	2.25	0.82 (0.06 to 1.58)
No. conversations about the health problems caused by e-cigarettes in past week	22	0.77 (1.74)	1.55 (1.47)	2.94	0.77 (0.23 to 1.32)
No. conversations about quitting e-cigarettes in past week	22	0.91 (1.63)	1.32 (1.52)	1.16	0.41 (−0.32 to 1.14)
E-cigarette quit intentions	19	1.56 (0.60)	1.91 (0.78)	2.54	0.35 (0.06 to 0.64)
No. of times forgoing an e-cigarette in past week	22	0.77 (0.96)	1.64 (2.51)	1.73	0.86 (−0.17 to 1.90)
Made e-cigarette quit attempt (for 24 h) in past week, %	21	5%	29%	--	24% (1% to 47%)

Response scale for worry and e-cigarette user prototypes ranged from 1 to 5, with 5 indicating higher endorsement. Response scale for e-cigarette quit intentions ranged from 1 to 4, with 4 indicating higher intentions. -- = not applicable. SD = standard deviation. CI = confidence interval.

**Table 5:** Recommended e-cigarette warning protocol.

Protocol Step	Rationale
1. Schedule weekly study visit appointments	A week between visits reduces participants’ financial burden of having to purchase refills and allows for frequent assessment of outcomes.
2. Determine e-cigarette consumption	Knowing how much an e-cigarette user typically consumes allows study staff to instruct the user on how many devices and refills to bring to the first and subsequent study visits.
3. Ask users to bring 1–2 e-cigarette devices and eight days’ worth of refills to study visits	Having users bring in their e-cigarette devices and refills on their own (rather than providing devices) prevents them from thinking that the study is giving users “free e-cigarettes.” Ask users to bring in their e-cigarette device and an eight-day supply of refills. If participants use more than one e-cigarette, ask them to bring in the two devices they own and use most frequently; if participants use disposable e-cigarettes, ask them to bring an eight-day supply of disposable e-cigarettes. Having users bring extra refills or disposable e-cigarettes allows for a buffer against missed appointments or in case they use more than expected in a given week.
4. Apply labels to users’ e-cigarette device(s) and refill(s)	Researchers label the devices and refills. Having researchers label devices and refills is likely to lead to higher protocol compliance. Apply labels to the e-cigarette on the area farthest from the mouth piece to minimize contact with saliva and increase chance of being seen during use. See Figure 4 for examples of recommended warning placement. Alternative approaches, such as users applying the warning labels to their own devices, may serve as an additional intervention component in addition to the warning itself.
5. Provide participation incentives for survey completion	Communicating that study incentives are for survey completion may reduce the possibility that participants will perceive that payments equate to receiving free e-cigarettes or refills.
6. Provide e-cigarette users materials about tobacco cessation services at study completion	Giving information about tobacco product cessation services at the end of the study may help users to quit using all tobacco products, if they have not already. It will also help explain that no tobacco product is safe to use, which may prevent unintended consequences such as compensatory cigarette smoking and correct any potentially inaccurate risk perceptions.

## Supplementary Materials

The following are available online at http://www.mdpi.com/1660-4601/15/8/1578/s1, Table S1: Study 1 measures, Table S2: Study 2 measures.

## References

[1] McMillen R.C., Gottlieb M.A., Shaefer R.M.W., Winickoff J.P., Klein J.D. (2015). Trends in electronic cigarette use among U.S. Adults: Use is increasing in both smokers and nonsmokers. Nicotine Tob. Res..

[2] Rass O., Pacek L.R., Johnson P.S., Johnson M.W. (2015). Characterizing use patterns and perceptions of relative harm in dual users of electronic and tobacco cigarettes. Exp. Clin. Psychopharmacol..

[3] Jamal A., King B.A., Neff L.J., Whitmill J., Babb S.D., Graffunder C.M. (2016). Current cigarette smoking among adults—United States, 2005–2015. Morb. Mortal. Wkly. Rep..

[4] Kasza K.A., Ambrose B.K., Conway K.P., Borek N., Taylor K., Goniewicz M.L., Cummings K.M., Sharma E., Pearson J.L., Green V.R. (2017). Tobacco-product use by adults and youths in the United States in 2013 and 2014. N. Engl. J. Med..

[5] Coleman B.N., Rostron B., Johnson S.E., Ambrose B.K., Pearson J., Stanton C.A., Wang B., Delnevo C., Bansal-Travers M., Kimmel H.L. (2017). Electronic cigarette use among US adults in the population assessment of tobacco and health (PATH) study, 2013–2014. Tob. Control.

[6] Pepper J.K., Brewer N.T. (2014). Electronic nicotine delivery system (electronic cigarette) awareness, use, reactions and beliefs: A systematic review. Tob. Control.

[7] McRobbie H., Bullen C., Hartmann-Boyce J., Hajek P. (2014). Electronic cigarettes for smoking cessation and reduction. Cochrane Database Syst. Rev..

[8] Rahman M.A., Hann N., Wilson A., Mnatzaganian G., Worrall-Carter L. (2015). E-cigarettes and smoking cessation: Evidence from a systematic review and meta-analysis. PLoS ONE.

[9] Grana R., Benowitz N., Glantz S.A. (2014). E-cigarettes: A scientific review. Circulation.

[10] Caraballo R.S., Shafer P.R., Patel D., Davis K.C., McAfee T.A. (2017). Quit methods used by US adult cigarette smokers, 2014–2016. Prev. Chronic Dis..

[11] Kalkhoran S., Glantz S.A. (2016). E-cigarettes and smoking cessation in real-world and clinical settings: A systematic review and meta-analysis. Respir. Med..

[12] Rutten L.J., Blake K.D., Agunwamba A.A., Grana R.A., Wilson P.M., Ebbert J.O., Okamoto J., Leischow S.J. (2015). Use of e-cigarettes among current smokers: Associations among reasons for use, quit intentions, and current tobacco use. Nicotine Tob. Res..

[13] Popova L., Ling P.M. (2013). Alternative tobacco product use and smoking cessation: A national study. Am. J. Public Health.

[14] Chen J., Bullen C., Dirks K. (2017). A comparative health risk assessment of electronic cigarettes and conventional cigarettes. Int. J. Environ. Res. Public Health.

[15] Farsalinos K.E., Polosa R. (2014). Safety evaluation and risk assessment of electronic cigarettes as tobacco cigarette substitutes: A systematic review. Ther. Adv. Drug Saf..

[16] National Academies of Sciences, Engineering, and Medicine (2018). Public Health Consequences of E-Cigarettes.

[17] Vardavas C.I., Anagnostopoulos N., Kougias M., Evangelopoulou V., Connolly G.N., Behrakis P.K. (2012). Short-term pulmonary effects of using an electronic cigarette: Impact on respiratory flow resistance, impedance, and exhaled nitric oxide. Chest.

[18] Glasser A.M., Collins L., Pearson J.L., Abudayyeh H., Niaura R.S., Abrams D.B., Villanti A.C. (2017). Overview of electronic nicotine delivery systems: A systematic review. Am. J. Prev. Med..

[19] Maina G., Castagnoli C., Passini V., Crosera M., Adami G., Mauro M., Filon F.L. (2016). Transdermal nicotine absorption handling e-cigarette refill liquids. Regul. Toxicol. Pharmacol..

[20] U.S. Food and Drug Administration, Health and Human Services (2016). Deeming tobacco products to be subject to the federal food, drug, and cosmetic act, as amended by the family smoking prevention and tobacco control act; restrictions on the sale and distribution of tobacco products and required warning statements for tobacco products. Final rule. Fed. Regist..

[21] Brewer N.T., Hall M.G., Noar S.M., Parada H., Stein-Seroussi A., Bach L.E., Hanley S., Ribisl K.M. (2016). Effect of pictorial cigarette pack warnings on changes in smoking behavior: A randomized clinical trial. JAMA Intern. Med..

[22] Noar S.M., Hall M.G., Brewer N.T. (2015). Pictorial cigarette pack warnings have important effects. Am. J. Public Health.

[23] Wackowski O., Hammond D., O’Connor R., Strasser A., Delnevo C. (2017). Considerations and future research directions for e-cigarette warnings—Findings from expert interviews. Int. J. Environ. Res. Public Health.

[24] Mays D., Smith C., Johnson A.C., Tercyak K.P., Niaura R.S. (2016). An experimental study of the effects of electronic cigarette warnings on young adult nonsmokers’ perceptions and behavioral intentions. Tob. Induc. Dis..

[25] Popova L., Ling P.M. (2014). Nonsmokers’ responses to new warning labels on smokeless tobacco and electronic cigarettes: An experimental study. BMC Public Health.

[26] Sanders-Jackson A., Schleicher N.C., Fortmann S.P., Henriksen L. (2015). Effect of warning statements in e-cigarette advertisements: An experiment with young adults in the united states. Addiction.

[27] Wackowski O.A., Hammond D., O’Connor R.J., Strasser A.A., Delnevo C.D. (2016). Smokers’ and e-cigarette users’ perceptions about e-cigarette warning statements. Int. J. Environ. Res. Public Health.

[28] Wackowski O.A., O’Connor R.J., Strasser A.A., Hammond D., Villanti A.C., Delnevo C.D. (2016). Smokers’ and e-cigarette users’ perceptions of modified risk warnings for e-cigarettes. Prev. Med. Rep..

[29] Brewer N.T., Hall M.G., Lee J.G., Peebles K., Noar S.M., Ribisl K.M. (2015). Testing warning messages on smokers’ cigarette packages: A standardised protocol. Tob. Control.

[30] Brewer N.T., Jeong M., Mendel J.R., Hall M.G., Zhang D., Parada H., Boynton M.H., Noar S.M., Baig S.A., Morgan J.C. (2018). Cigarette pack messages about toxic chemicals: A randomised clinical trial. Tob. Control.

[31] Berinsky A.J., Huber G.A., Lenz G.S. (2012). Evaluating online labor markets for experimental research: Amazon.Com’s Mechanical Turk. Political Anal..

[32] Buhrmester M., Kwang T., Gosling S.D. (2011). Amazon’s Mechanical Turk: A new source of inexpensive, yet high-quality, data?. Perspect. Psychol. Sci..

[33] Paolacci G., Chandler J., Ipeirotis P. (2010). Running experiments on amazon Mechanical Turk. Judgm. Decis. Mak..

[34] Rand D.G. (2012). The promise of Mechanical Turk: How online labor markets can help theorists run behavioral experiments. J. Theor. Biol..

[35] Jeong M., Zhang D., Morgan J.C., Cornacchione J., Osman A., Boynton M.H., Mendel J.R., Brewer N.T. (2008). Similarities and differences in tobacco control research findings from convenience and probability samples. Ann. Behav. Med..

[36] Centers for Disease Control and Prevention (2008). Adult Tobacco Survey (ATS).

[37] R Core Team (2000). R Language Definition.

[38] Brewer N.T., Parada H., Hall M.G., Boynton M.H., Noar S.M., Ribisl K.M. (2018). Understanding why pictorial cigarette pack warnings increase quit attempts. Ann. Behav. Med..

[39] Nonnemaker J., Farrelly M., Kamyab K., Busey A., Mann N. (2010). Experimental Study of Graphic Cigarette Warning Labels: Final Results Report.

[40] Hall M.G., Peebles K., Bach L.E., Noar S.M., Ribisl K.M., Brewer N.T. (2015). Social interactions sparked by pictorial warnings on cigarette packs. Int. J. Environ. Res. Public Health.

[41] Morgan J.C., Southwell B.G., Noar S.M., Ribisl K.M., Golden S.D., Brewer N.T. (2017). Frequency and content of conversations about pictorial warnings on cigarette packs. Nicotine Tob. Res..

[42] Borland R., Yong H., Wilson N., Fong G.T., Hammond D., Cummings K.M., Hosking W., McNeill A. (2009). How reactions to cigarette packet health warnings influence quitting: Findings from the ITC Four-Country survey. Addiction.

[43] Hammond D., Fong G.T., McDonald P.W., Cameron R., Brown K.S. (2003). Impact of the graphic Canadian warning labels on adult smoking behaviour. Tob. Control.

[44] Klein W.M., Zajac L.E., Monin M.M. (2009). Worry as a moderator of the association between risk perceptions and quitting intentions in young adult and adult smokers. Ann. Behave. Med..

[45] Borland R., Hill D. (1997). Initial impact of the new Australian tobacco health warnings on knowledge and beliefs. Tob. Control.

[46] Li L., Borland R., Fong G.T., Jiang Y., Yang Y., Wang L., Partos T.R., Thrasher J.F. (2015). Smoking-related thoughts and microbehaviours, and their predictive power for quitting: Findings from the International Tobacco Control (ITC) China Survey. Tob. Control.

[47] (2014). PATH: Population Assessment of Tobacco and Health. http://www.pathstudyinfo.nih.gov/UI/HomeMobile.aspx.

[48] Dijkstra A., Brosschot J. (2003). Worry about health in smoking behaviour change. Behav. Res. Ther..

[49] Ranby K.W., Lewis M.A., Toll B.A., Rohrbaugh M.J., Lipkus I.M. (2013). Perceptions of smoking-related risk and worry among dual-smoker couples. Nicotine Tob. Res..

[50] Magnan R.E., Koblitz A.R., Zielke D.J., McCaul K.D. (2009). The effects of warning smokers on perceived risk, worry, and motivation to quit. Ann. Behav. Med..

[51] Magnan R.E., Koblitz A.R., McCaul K.D., Dillard A.J. (2013). Self-monitoring effects of ecological momentary assessment on smokers’ perceived risk and worry. Psychol. Assess..

[52] McCool J., Cameron L., Petrie K. (2004). Stereotyping the smoker: Adolescents’ appraisals of smokers in film. Tob. Control.

[53] McCool J., Cameron L.D., Robinson E. (2011). Do parents have any influence over how young people appraise tobacco images in the media?. J. Adolesc. Health.

[54] Pepper J.K., Cameron L.D., Reiter P.L., McRee A.L., Brewer N.T. (2013). Non-smoking male adolescents’ reactions to cigarette warnings. PLoS ONE.

[55] Morey R.D., Rouder J.N., Jamil T. (2015). Bayesfactor: Computation of Bayes Factors for Common Designs.

[56] Kruschke J.K. (2011). Bayesian assessment of null values via parameter estimation and model comparison. Perspect. Psychol. Sci..

[57] Kruschke J.K. (2013). Bayesian estimation supersedes the t test. J. Exp. Psychol. Gen..

[58] McNeish D. (2016). On using bayesian methods to address small sample problems. Struct. Equ. Model..

[59] Jamil T., Ly A., Morey R.D., Love J., Marsman M., Wagenmakers E.J. (2017). Default “gunel and dickey” bayes factors for contingency tables. Behav. Res. Methods.

[60] Morey R.D., Rouder J.N. (2011). Bayes factor approaches for testing interval null hypotheses. Psychol. Methods.

[61] Hajek P., Etter J.-F., Benowitz N., Eissenberg T., McRobbie H. (2014). Electronic cigarettes: Review of use, content, safety, effects on smokers and potential for harm and benefit. Addiction.

[62] Majeed B.A., Weaver S.R., Gregory K.R., Whitney C.F., Slovic P., Pechacek T.F., Eriksen M.P. (2017). Changing perceptions of harm of e-cigarettes among U.S. Adults, 2012–2015. Am. J. Prev. Med..

