# Supplementary material for: Placing Health Warnings on E-Cigarettes: A Standardized Protocol
Source: Int J Environ Res Public Health. 2018 Jul 25;15(8):1578. doi: 10.3390/ijerph15081578 (PMC6122039; doi:10.3390/ijerph15081578)
Supplement: Supplementary file 1 [file ijerph-15-01578-s001.pdf]

International Journal of
*Environmental Research and Public Health*

MDPI

# Supplementary Materials: Placing Health Warnings on E-Cigarettes: A Standardized Protocol

**Table S1.** Study 1 measures.

| Construct | Item | Response scale | Source |
|---|---|---|---|
| Prompt | The next question is about electronic cigarettes or e-cigarettes. You may also know them as vape-pens, hookah-pens, e-hookahs, or e-vaporizers. Some look like cigarettes, and others look like pens or small pipes. These are battery-powered, usually contain liquid nicotine, and produce vapor instead of smoke. | | New item |
| E-cigarette use | Have you EVER used an e-cigarette EVEN ONE TIME?<br>[If Yes, then answer next item. If No, then skip] | 1=Yes<br>2=No | New item, adapted from PATH |
| E-cigarette use | Do you NOW use e-cigarettes every day, some days, or not at all? | 1=Every day<br>2=Some days<br>3=Not at all | New item, adapted from PATH |
| E-cigarette use frequency | Think about the past week. On average, on how many days did you use an e-cigarette? | 0=0 days<br>1=1 day<br>2=2 days<br>...<br>7=7 days | New item, adapted from PATH |
| E-cigarette use intensity | On days when you used an e-cigarette, about how many puffs did you take? | # of puffs: _____ | New item, adapted from PATH |
| No. of devices | How many different e-cigarette devices do you own <u>and</u> use regularly? This includes vape pens, mods, and other vaping devices. | 0=None<br>1=1 device<br>2=2 devices<br>…<br>7=7 or more devices | New item |
| Type of refills | In the past 30 days, what refills did you use for your e-cigarette? Check all that apply. | 1=E-liquid that I pour in the tank<br>2=Pre-filled cartridges<br>3=I use e-cigarettes that are not refillable (I throw them away when I'm done)<br>4=Other (specify) ______ | New item |
| Willing to have device & refill labeled | **E-cigarette health warning study**<br><br>Imagine you have been recruited to participate in a new research study. In this study, you would come to the research office every week for 4 weeks, and a sticker with a health warning would be put on your e-cigarette. During the study, you'd use the labeled e-cigarette as you normally would. You would get up to $300 for being in the study.<br><br>As part of the study, would you be willing to have a warning sticker on your…<br><br>1. E-cigarette<br>2. Package your e-cigarette came in<br>3. Refills (e-liquid bottles or cartridges)<br>4. Package for e-liquids or cartridges | 1=Yes<br>2=Not sure<br>0=No | New item |
| Able to bring in 8-day supply | Do you think you would be able to bring 8 days' worth of e-cigarette refills to each appointment? This would be | 1=Yes<br>2=Not sure<br>0=No | New item |

| | | | |
|---|---|---|---|
| | whatever you usually use, such as e-liquid bottles, cartridges or disposable e-cigarettes. | 3=Does not apply: I only use other people's e-cigarettes | |
| E-cigarette quit attempt | In the past week, did you stop using your e-cigarette for 1 day or longer because you were trying to quit using e-cigarettes? | 1=Yes<br>0=No | Adapted from CDC ATS, 2008 |
| Age | How old are you? | 1=18 to 24 years old<br>2=25 to 29 years old<br>3=30 to 44 years old<br>4=45 to 59 years old<br>5=60 years old or older | Adapted from 2010 Census |
| Sex | What sex were you assigned at birth, on your original birth certificate? Was it ... | 1=Male<br>2=Female | Modified Williams Institute item, 2009 |
| Gender identity | Do you describe yourself as ... | 1=Male<br>2=Female<br>3-Transgender<br>4= Other (please specify) | Modified Williams Institute item, 2009 |
| Sexual orientation | The next question is about your sexual orientation. Do you consider yourself to be ... | 1=Straight or heterosexual<br>2=Gay or lesbian<br>3=Bisexual | Adapted from Williams Institute item, 2009 |
| Ethnicity | Are you of Hispanic, Latino, or Spanish origin? | 0=No<br>1=Yes | Adapted from 2010 Census |
| Race | Which one of these groups would you say best represents your race? | 1=White<br>2=Black or African American<br>3=American Indian or Alaska Native<br>4=Asian<br>5=Pacific Islander<br>6= Other (please specify) | Adapted from 2013 BRFSS |
| Education | What is the highest degree or level of school you have completed? | 1=Less than high school degree<br>2=High school graduate (or GED)<br>3=Some college or technical school<br>4=Associate's degree<br>5=Bachelor's degree<br>6=Graduate or professional degree | Adapted from 2010 Census |
| Cigarette use | Have you smoked at least 100 cigarettes in your entire life? | 1=Yes<br>0=No | Adapted from 2013 BRFSS |
| Cigarette use | Do you now smoke cigarettes every day, some days or not at all? | 2=Every day<br>1=Some days<br>0=Not at all | Adapted from 2013 BRFSS and PATH |

**Table S2.** Study 2 measures.

| Construct | Item | Response scale | Source |
|---|---|---|---|
| Prompt | First we are going to ask you about e-cigarettes and other vaping devices, such as vape pens and mods. These devices are battery-powered and produce vapor instead of smoke. Some have nicotine in the liquid, others do not. We will call these devices "e-cigarettes" in this survey. | | New item |
| E-cigarette use | Think about the past week. On average, on how many days did you use an e-cigarette? | 0=0 days<br>1=1 day<br>2=2 days<br>…<br>7=7 days | New item, adapted from PATH |
| E-cigarette use | On days when you used an e-cigarette, about how many puffs did you take? | ____ puffs | New item, adapted from PATH |
| Exposure | Think about the past week. When you used an e-cigarette, how much of the time did you use the e-cigarette we labeled? (Circle a number) | [Number line 0 -100%<br>0= labeled None of the time<br>50= labeled Half of the time<br>100= labeled All of the time] | New item |
| Quit attempt | In the past week, did you stop using your e-cigarette for 1 day or longer because you were trying to quit using e-cigarettes? | 1= Yes<br>0= No | New item |
| Process | Overall, how difficult or easy was it to be in this study? | 1=Very difficult<br>2=Difficult<br>3=Neither difficult nor easy<br>4=Easy<br>5=Very easy | Adapted from Brewer et al., 2015 |
| Process | How difficult or easy was it to bring in your e-cigarette each week? | 1=Very difficult<br>2=Difficult<br>3=Neither difficult nor easy<br>4=Easy<br>5=Very easy | Adapted from Brewer et al., 2015 |
| Process | How difficult or easy was it to bring in your refills each week? | 1=Very difficult<br>2=Difficult<br>3=Neither difficult nor easy<br>4=Easy<br>5=Very easy<br>6=Not applicable | Adapted from Brewer et al., 2015 |
| Process | If you had the chance, would you participate in this study again? | 1=Definitely not<br>2=Probably not<br>3=Maybe<br>4=Probably yes<br>5=Definitely yes | Adapted from Brewer et al., 2015 |
| Process | Would you recommend this study to a friend? | 1=Definitely not<br>2=Probably not<br>3=Maybe<br>4=Probably yes<br>5=Definitely yes | Adapted from Brewer et al., 2015 |
| Prompt | The next questions are about the warning label we put on your e-cigarette. | | |
| Process | How easy was the label to read? | 1=Not at all<br>2=A little<br>3=Somewhat<br>4=Very<br>5=Extremely | New item |
| Negative affect | How much did the warning on your e-cigarette make you feel… | 1=Not at all<br>2=A little<br>3=Somewhat | Adapted from Nonnemaker et al., 2010 |

| | | | |
|---|---|---|---|
| | Anxious? | 4=Very<br>5=Extremely | |
| Negative affect | How much did the warning on your e-cigarette make you feel…<br><br>Sad? | 1=Not at all<br>2=A little<br>3=Somewhat<br>4=Very<br>5=Extremely | Adapted from Watson, 1988 |
| Negative affect | How much did the warning on your e-cigarette make you feel…<br><br>Scared? | 1=Not at all<br>2=A little<br>3=Somewhat<br>4=Very<br>5=Extremely | Adapted from Nonnemaker et al., 2010, and Watson, Clark, and Tellegen (1988) |
| Negative affect | How much did the warning on your e-cigarette make you feel…<br><br>Guilty? | 1=Not at all<br>2=A little<br>3=Somewhat<br>4=Very<br>5=Extremely | Adapted from Nonnemaker 2010 and Keller and Block, 1996, and Watson, Clark, and Tellegen, 1988 |
| Negative affect | How much did the warning on your e-cigarette make you feel…<br><br>Disgusted? | 1=Not at all<br>2=A little<br>3=Somewhat<br>4=Very<br>5=Extremely | Adapted from Nonnemaker et al., 2010 |
| Prompt | Say how much you agree or disagree with the next statements about the warning on your e-cigarette. | | |
| Perceived effectiveness | The warning makes me concerned about the health effects of using e-cigarettes. | 1=Strongly disagree<br>2=Somewhat disagree<br>3=Neither agree nor disagree<br>4=Somewhat agree<br>5=Strongly agree | Brewer et al., 2018 and Baig et al., (under review) |
| Perceived effectiveness | The warning makes using e-cigarettes seem unpleasant to me. | 1=Strongly disagree<br>2=Somewhat disagree<br>3=Neither agree nor disagree<br>4=Somewhat agree<br>5=Strongly agree | Brewer et al., 2018 and Baig et al., (under review) |
| Perceived effectiveness | The warning discourages me from wanting to use e-cigarettes. | 1=Strongly disagree<br>2=Somewhat disagree<br>3=Neither agree nor disagree<br>4=Somewhat agree<br>5=Strongly agree | Brewer et al., 2018 and Baig et al., (under review) |
| Cognitive elaboration | When you noticed your e-cigarette, how often did you think about the information that the warning conveys? | 1=Never<br>2=Rarely<br>3=Sometimes<br>4=Often<br>5=All the time | Adapted from Hammond et al., 2003 |
| Cognitive elaboration | How much did the warning cause you to think about the harmful effects of using e-cigarettes? | 1=Not at all<br>2=A little bit<br>3=Somewhat<br>4=Quite a bit<br>5=Very much | Adapted from Borland et al., 2009 |
| Attention/noticing | In the past week, how often did you notice the warning? | 1=Never<br>2=Rarely<br>3=Sometimes<br>4=Often<br>5=All the time | Adapted from Nonnemaker et al., 2010 |

| | | | |
|---|---|---|---|
| Attention/noticing | In the past week, how often did you read or look closely at the warning? | 1=Never<br>2=Rarely<br>3=Sometimes<br>4=Often<br>5=All the time | Adapted from Nonnemaker et al., 2010 |
| Cognitive elaboration | When your e-cigarette is not in sight, how often do you think about the message that the warning conveys? | 1=Never<br>2=Rarely<br>3=Sometimes<br>4=Often<br>5=All the time | Adapted from Hammond et al., 2003 |
| Prompt | The next questions are about talking with other people. | | |
| Social interactions | Who did you talk to about the warning? (Check all that apply) | 1=My spouse or significant other<br>2=My child<br>3=Other family member<br>4=Friend<br>5=Co-worker<br>6=Health care provider<br>7=Someone I did not previously know<br>8=Other<br>9=I did not talk to anyone about the warning | Adapted from Hall et al. (2015) and Morgan et al. (In press) |
| Social interactions | Think about the conversations you had about the warning. Who did you talk to <u>the most</u> about the warning? | 1=My spouse or significant other<br>2=My child<br>3=Other family member<br>4=Friend<br>5=Co-worker<br>6=Health care provider<br>7=Someone I did not previously know<br>8=Other | Adapted from Hall et al. (2015) and Morgan et al. (In press) |
| Social interactions | Think about the conversations you had about the warning. What came up during these conversations? (Check all that apply) | 1=The health problems caused by using e-cigarettes<br>2=The chemicals in e-cigarette vapor<br>3=The information in this warning is new to me<br>4=The warning makes me want to quit using e-cigarettes<br>5=The warning would make other e-cigarette users want to quit using e-cigarettes<br>6=The warning would stop people from starting to use e-cigarettes<br>7=The warning should be on e-cigarettes<br>8=Made fun of the warning<br>9=This research study, specify: ______<br>10=None of the above<br>11=Other: ______ | Adapted from Hall et al., 2015 and Morgan et al., (In press) |

| | | | |
|---|---|---|---|
| Social interactions | Think about the conversations you had about the warning. Would you say that in general these conversations were… | 1=Mostly positive<br>2=Mostly negative<br>3=Somewhere in between | New item |
| Social interactions | Think about the conversations you had about the warning. Did you or the other person say that the message made you feel… (Check all that apply) | 1=Anxious<br>2=Sad<br>3=Scared<br>4=Guilty<br>5=Disgusted<br>6=None of the above | Adapted from Hall et al., 2015 and Morgan et al., (In press) |
| Social interactions | In the past week, how many times did you talk to others about the <u>health problems</u> caused by using e-cigarettes? | ______ times | Adapted from Hall et al., 2015 and Morgan et al., (In press) |
| Social interactions | In the past week, how many times did you talk to others about the <u>addictiveness</u> of e-cigarettes? | ______ times | Adapted from Hall et al., 2015 and Morgan et al., (In press) |
| Social interactions | In the past week, how many times did you talk to others about <u>quitting</u> e-cigarettes? | ______ times | Adapted from Hall et al., 2015 and Morgan et al., (In press) |
| Reactance | Please say how much you agree or disagree with each statement below about the warning we put on your e-cigarette and refills.<br><br>This warning is trying to manipulate me. | 1=Strongly disagree<br>2=Somewhat disagree<br>3=Neither agree nor disagree<br>4=Somewhat agree<br>5=Strongly agree | Hall et al., 2017 |
| Reactance | This warning annoys me. | 1=Strongly disagree<br>2=Somewhat disagree<br>3=Neither agree nor disagree<br>4=Somewhat agree<br>5=Strongly agree | Hall et al., 2017 |
| Reactance | This warning is overblown. | 1=Strongly disagree<br>2=Somewhat disagree<br>3=Neither agree nor disagree<br>4=Somewhat agree<br>5=Strongly agree | Hall et al., 2017 |
| Worry | How worried are you about your health because of your e-cigarette use? | 1=Not at all<br>2=A little<br>3=Fairly<br>4=Very<br>5=Extremely | Modified from Dijkstra, 2003 and Ranby, 2013 |
| Worry | How anxious do you feel when you think of the possible consequences of your e-cigarette use? | 1=Not at all<br>2=A little<br>3=Fairly<br>4=Very<br>5=Extremely | Modified from Dijkstra, 2003 and Ranby, 2013 |
| Worry | How worried are you that your e-cigarette use bothers people who don't use e-cigarettes? | 1=Not at all<br>2=A little<br>3=Fairly<br>4=Very<br>5=Extremely | Adapted from Magnan, 2009 and Magnan, 2013 |
| Worry | How worried are you that your e-cigarette use can be harmful to other people? | 1=Not at all<br>2=A little<br>3=Fairly<br>4=Very<br>5=Extremely | Adapted from Magnan, 2009 and Magnan, 2013 |
| Ecig user prototypes (prompt) | How much do the following characteristics describe a typical e-cigarette user your age? | | Adapted from McCool et al., 2004, McCool et al., 2011, |

| | | | |
|---|---|---|---|
| | | | and Pepper et al., 2013 |
| Ecig user prototypes | Cool | 1=Not at all<br>2=A little bit<br>3=Somewhat<br>4=Quite a bit<br>5=Very much | " |
| Ecig user prototypes | Disgusting | 1=Not at all<br>2=A little bit<br>3=Somewhat<br>4=Quite a bit<br>5=Very much | " |
| Ecig user prototypes | Healthy | 1=Not at all<br>2=A little bit<br>3=Somewhat<br>4=Quite a bit<br>5=Very much | " |
| Ecig user prototypes | Smart | 1=Not at all<br>2=A little bit<br>3=Somewhat<br>4=Quite a bit<br>5=Very much | " |
| Ecig user prototypes | Sexy | 1=Not at all<br>2=A little bit<br>3=Somewhat<br>4=Quite a bit<br>5=Very much | " |
| Ecig user prototypes | Unattractive | 1=Not at all<br>2=A little bit<br>3=Somewhat<br>4=Quite a bit<br>5=Very much | " |
| Ecig user prototypes | Immature | 1=Not at all<br>2=A little bit<br>3=Somewhat<br>4=Quite a bit<br>5=Very much | " |
| Ecig user prototypes | Inconsiderate | 1=Not at all<br>2=A little bit<br>3=Somewhat<br>4=Quite a bit<br>5=Very much | " |
| Quit intentions | How likely are you to quit using e-cigarettes in the next month? | 1=Not at all likely<br>2=A little likely<br>3=Somewhat likely<br>4=Very likely | Adapted from Klein et al., 2009 |
| Quit intentions | How much do you plan to quit using e-cigarettes in the next month? | 1=Not at all<br>2=A little<br>3=Somewhat<br>4=Very much | Adapted from Klein et al., 2009 |
| Quit intentions | How interested are you in quitting e-cigarettes in the next month? | 1=Not at all interested<br>2=A little interested<br>3=Somewhat interested<br>4=Very interested | Adapted from Klein et al., 2009 |
| Forgoing an e-cigarette | In the past week, how often have you stopped yourself from using an e-cigarette because you wanted to cut back? | 1=Never<br>2=1-2 times<br>3=3-4 times<br>4=5-9 times<br>5=10 or more times | Adapted from Borland & Hill, 1997) and Li, 2014 |

| | | | |
|---|---|---|---|
| Prompt | The next questions are about cigarettes. | | |
| Cigarette use | Have you smoked at least 100 cigarettes in your entire life? | 1=Yes<br>0=No | BRFSS |
| Cigarette use | Do you now smoke cigarettes every day, some days or not at all? | 2=Every day<br>1=Some days<br>0=Not at all | BRFSS |
| Smoking frequency | On how many of the past 7 days did you smoke cigarettes? | 0=0 days<br>1=1 day<br>2=2 days<br>3=3 days<br>4=4 days<br>5=5 days<br>6=6 days<br>7=7 days | PATH, 2014 |
| Smoking quantity | On average, on the days you smoked, how many cigarettes did you usually smoke <u>each day</u>? A pack usually has 20 cigarettes in it. | ____ cigarettes <u>per day</u><br>□ Does not apply to me. I did not smoke. | Modified PATH, 2014 |
| Prompt | The next questions will help us understand the diverse population in this study. Your survey responses are completely confidential. | | New item |
| Sex | What sex were you assigned at birth, on your original birth certificate? | 1=Male<br>2=Female | Adapted from Williams Institute, 2009 |
| Gender identity | How do you describe your gender identity? | 1=Male<br>2=Female<br>3=Transgender<br>4=Other gender identity, specify: _________ | Adapted from Williams Institute, 2009 |
| Sexual orientation | The next question is about your sexual orientation. Do you consider yourself to be… | 1=Straight or heterosexual<br>2=Gay or lesbian<br>3=Bisexual | Adapted from Williams Institute, 2009 |
| Age | How old are you? | ____ years old | Adapted from 2010 Census |
| Ethnicity | Are you of Hispanic, Latino or Spanish origin? | 1=Yes<br>2=No | Adapted from 2010 Census |
| Race | What is your race? (Check all that apply) | 1=White<br>2=Black or African American<br>3=American Indian or Alaska Native<br>4=Asian<br>5=Native Hawaiian or Other Pacific Islander<br>6=Some other race, specify: __________ | Adapted from 2013 BRFSS |
| Education | What is the highest degree or level of school you have completed? | 1=Less than high school degree<br>2=High school graduate (or GED)<br>3=Some college or technical school<br>4=Associate's degree<br>5=Bachelor's degree<br>6=Graduate or professional degree | Adapted from 2010 Census |
| Income | Which of the following categories best describes your total household income in the past 12 months? | 1=Less than $10,000<br>2=$10,000 to $14,999<br>3=$15,000 to $24,999 | Adapted from 2010 Census and 2013 BRFSS |

| | | | |
|---|---|---|---|
| | | 4=$25,000 to $34,999<br>5=$35,000 to $49,999<br>6=$50,000 to $74,999<br>7=$75,000 to $99,999<br>8=$100,000 to $149,999<br>9=$150,000 to $199,999<br>10=$200,000 or more | |
